# Effects of three pulmonary ventilation regimes in patients undergoing coronary artery bypass graft surgery: a randomized clinical trial

**DOI:** 10.1038/s41598-021-86281-4

**Published:** 2021-03-24

**Authors:** Revati Amin, Gopala Krishna Alaparthi, Stephen R. Samuel, Kalyana Chakravarthy Bairapareddy, Harish Raghavan, K. Vaishali

**Affiliations:** 1 Department of Physiotherapy, Kasturba Medical College, Manipal Academy of Higher Education, Bejai, Mangalore, 575004 India; 2 Department of Physiotherapy, College of Health Sciences, University of Sharjah, Sharjah, United Arab Emirates; 3 Department of Cardiothoracic Surgery, Kasturba Medical Hospital, Mangalore, 575004 India; 4 Department of Physiotherapy, Manipal College of Health Professions, Manipal Academy of Higher Education, Manipal, 576104 India

**Keywords:** Health care, Medical research

## Abstract

The aim was to compare the effect of diaphragmatic breathing exercise (DBE), flow- (FIS) and volume-oriented incentive spirometry (VIS) on pulmonary function- (PFT), functional capacity-6-Minute Walk Test (6 MWT) and Functional Difficulties Questionnaire (FDQ) in subjects undergoing Coronary Artery Bypass Graft surgery (CABG). The purpose of incorporating pulmonary ventilator regimes is to improve ventilation and avoid post-operative pulmonary complications. CABG patients (n = 72) were allocated to FIS, VIS and DBE groups (n = 24 each) by block randomization. Preoperative and postoperative values for PFT were taken until day 7 for all three groups. On 7th postoperative day, 6 MWT and FDQ was analyzed using ANOVA and post-hoc analysis. PFT values were found to be decreased on postoperative day 1(Forced Vital Capacity (FVC) = FIS group—65%, VIS group—47%, DBE group—68%) compared to preoperative day (p < 0.001). PFT values for all 3 groups recovered until postoperative day 7 (FVC = FIS group—67%, VIS group—95%, DBE group—59%) but was found to reach the baseline in VIS group (p < 0.001). When compared between 3 groups, statistically significant improvement was observed in VIS group (p < 0.001) in 6 MWT and FDQ assessment. In conclusion, VIS was proven to be more beneficial in improving the pulmonary function (FVC), functional capacity and FDQ when compared to FIS and DBE.

## Introduction

In spite of advances in surgical techniques and amelioration of the condition of patients through the preoperative and postoperative care associated with cardiac surgeries, morbidity and mortality due to development of pulmonary complications is relatively high. Coronary artery bypass graft (CABG) surgeries are often associated with pulmonary dysfunction, such as atelectasis, restrictive ventilatory disorder and hypoxemia^1^. The ability to perform periodic deep inspiration and cough effectively, to prevent accumulation of secretions and improved gas exchange^2^, is adversely affected by pain and postoperative apprehension associated with changes in lung mechanics resulting from surgery^3^.

For the prevention and treatment of postoperative pulmonary complications, respiratory physiotherapy is routinely used, after cardiac surgery. The main aim of physiotherapy is to improve ventilation–perfusion matching, increase lung volume, enhance mucociliary clearance and decrease pain^3,4^.

Postoperative physiotherapy techniques include many techniques such as early mobilization, positioning, breathing exercises, splinted coughing or huffing, percussion, vibration, active cycle of breathing techniques (ACBT) and also the use of different mechanical devices such as the incentive spirometer (IS), positive expiratory pressure mask therapy and continuous positive airway pressure^4–6^.

Diaphragmatic breathing exercises (DBE) are performed to initiate diaphragmatic descent during inhalation and diaphragmatic ascent during exhalation. When performing diaphragmatic breathing the patient inhales, the air reaches the alveoli, it reverses the post-operative hypoxemia which is the result of the anesthesia, improves the ventilation and oxygenation, reduces the work of breathing as the muscles of the neck and the shoulders relax, and also increases the excursion of the diaphragm^5^. Reanult et al. provided 3 sets of 10 breaths to bring about an effect in the diaphragmatic breathing exercise group in his study^3^.

**Figure 1:**
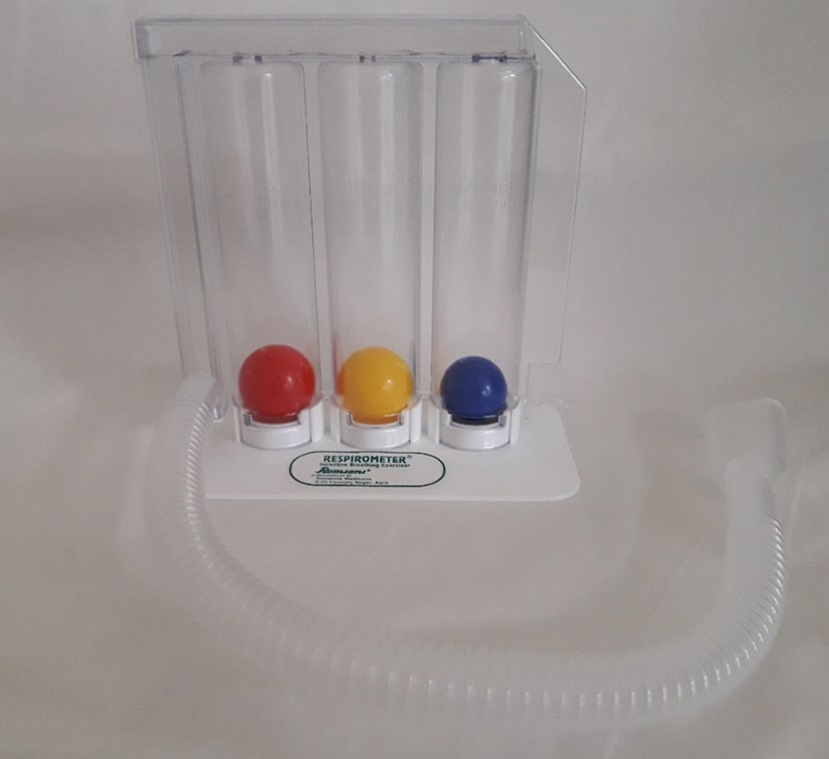
Flow-oriented incentive spirometer (Triflow Device).

Incentive spirometry (IS) is a mechanical breathing device in which the patient is expected to take long, slow deep breaths imitating natural sighing which also gives a positive visual feedback. Incentive spirometers are accessible either by volume of inspiration (volume-oriented) or flow rate (flow-oriented) (Fig. 1)^5^.

**Figure 2:**
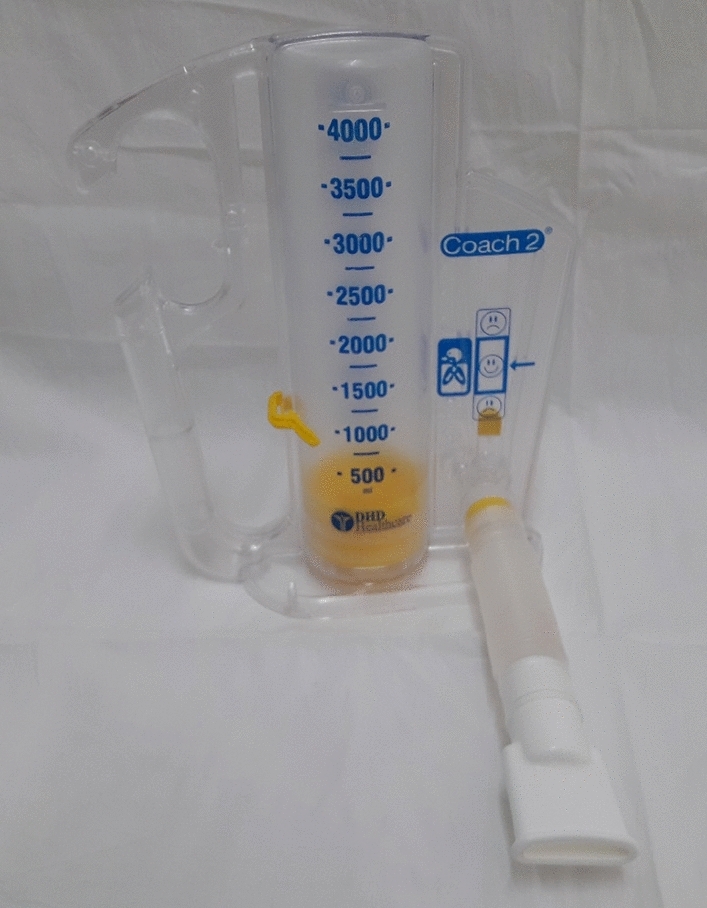
Volume-oriented incentive spirometer (Coach 2 Device).

A typical flow-oriented Incentive Spirometer (FIS) (Triflow device) consists of three chambers in a series each of which consists of a ball. The ball rises in the chamber as the patient sucks in the air and generates a sub-atmospheric pressure above the ball. When an inspiratory flow of 600 mL/s is achieved then the ball in the first chamber rises, an inspiratory flow of 900 mL/s raises the ball in the second column and an inspiratory flow of 1200 mL/s raises the ball in the third chamber (Fig. 2)^5,7,8^.

A typical volume-oriented Incentive Spirometer (VIS) is a compact device of 4000 mL capacity and has a one-way valve to prevent exhalation into the unit, it consists of a corrugated large-bore breathing hose and mouthpiece that connects the patient to a flexible plastic bellow. When the patient inspires in through the breathing hose, the bellow raises, which indicates the volumetric displacement via an indicator present on the device enclosure. On obtaining its maximum displacement for a given breath, the patient is requested to hold the bellow in the same position for 5–10 s (i.e. the end-inspiratory hold). After termination of the manoeuvre, the patient removes the mouthpiece, enabling gravity to return the bellow to its original position^5,7,8^.

Studies have suggested that there is a significant physiological variability in the effect of the flow and volume-oriented incentive spirometer. Flow-oriented devices (Triflow device) causes increased work of breathing and increases the muscular activity of the upper chest. Whereas volume-oriented devices (Coach 2 device) enforces less work of breathing and also improves the activity of the diaphragm^5,9–14^. In a study conducted by Renault et al., the dosage provided to the incentive spirometry group was 3 sets of 10 breaths each to bring about an effect^3^.

Thoracic mobility for patients post cardiac surgery is reduced to a great extent which affects most of their activities of daily living. This can be assessed using the Functional Difficulty Questionnaire (FDQ). FDQ was evolved based on findings obtained from the literature, clinical practice, and kinematics. The FDQ is comprised of 13 different functional tasks, which are imperative to everyday living and include movements associated with the thoracic region. FDQ has good internal consistency (Cronbach alpha coefficient = 0.971), excellent test–retest reliability (intraclass correlation 0.918). The MDC is 16.35 cm out 130 cm^9^.

In postoperative respiratory care, as a part of the routine prophylactic and therapeutic regimen various chest physiotherapy techniques are used clinically. In subjects who undergo coronary artery bypass graft surgery, there is ambiguity in evidence which compares the efficacy of diaphragmatic breathing, flow-oriented and volume-oriented incentive spirometry in pulmonary function (forced vital capacity—FVC), functional capacity and Functional Difficulties Questionnaire (FDQ). The present study aims (a) to compare the pre-operative and post-operative effects of DBE, FIS and VIS on the pulmonary function (FVC), (b) to compare the post-operative effects of DBE, FIS and VIS on the functional capacity (6 MWT) and functional difficulty questionnaire (FDQ) in subjects undergoing CABG surgery.

## Results

A total of 72 patients who were posted for Coronary Artery Bypass Graft surgery meeting the inclusion criteria were selected for the study with 24 patients in each intervention group. Baseline demographic characteristics of the participants such as age, height, weight, BMI, risk factors, type of surgery and duration of surgery are presented in Table 1. There were no statistically significant differences between the groups.

**Table 1:** Demographic characteristics of subjects undergoing coronary artery bypass graft surgery in all three-intervention groups.

Variables	Diaphragmatic breathing exercise group (DB) (N = 24)	Flow-incentive spirometry group (FS) (N = 24)	Volume-incentive spirometry group (VS) (N = 24)	p value (p < 0.05)
Age (mean ± SD)	60.25 ± 9.32	61.89 ± 10.34	65.54 ± 9.91	0.170
Gender (M:F)	18:6	19:5	17:7	
Height (cm) (mean ± SD)	157.17 ± 6.30	155.46 ± 6.64	155.58 ± 8.14	0.649
Weight (kg) (mean ± SD)	74.79 ± 10.00	71.92 ± 10.59	71.25 ± 10.11	0.448
BMI (mean ± SD)	30.33 ± 4.09	29.67 ± 3.41	29.48 ± 4.11	0.730
Surgery type (off pump:on pump)	18:6	19:5	18:6	
Smokers (n)	11	15	12	
Active smokers (1–5 years:5–10 years) (n)	3:2	3:3	2:5	
Quit smoking (1–5 years:5–10 years) (n)	3:3	4:5	3:2	
Duration of surgery (h)	1.78 ± 0.67	1.89 ± 0.59	1.76 ± 0.66	0.63
Hypertension (n)	14	20	11	
Diabetes (n)	19	12	15	
Coronary artery lesions (single:double:triple) vessel disease	10:8:6	20:2:2	12:8:4	
Bronchodilators (salbutamol:ipratropium bromide) (n)	(4:3)	(3:3)	(2:4)	
SPO_2_ levels post 6MWT (mean ± SD)	90 ± 7.00	92 ± 6.33	92 ± 7.45	
Pain: visual analog scale (VAS)	4 ± 3	5 ± 2	4 ± 3	
**ABG: (mean ± SD)**				
PaO_2_	98 ± 0.81	98.66 ± 0.47	97.66 ± 0.94	
PaCO_2_	33.33 ± 1.24	32.66 ± 0.47	33.33 ± 0.47	
pH	7.27 ± 0.001	7.28 ± 0.002	7.37 ± 0.07	
Base excess	− 2.5 ± 0.08	− 2.5 ± 0.08	− 2.4 ± 0.08	

**Figure 3:**
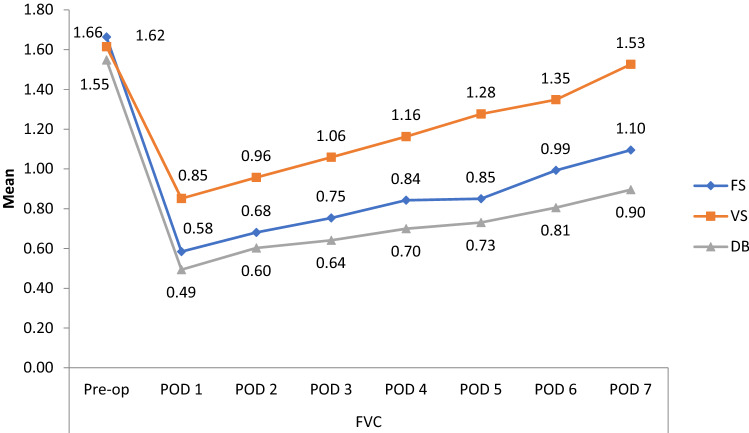
Comparison of forced vital capacity (FVC) before and after coronary artery bypass graft surgery between three groups.

Forced vital capacity (FVC) was compared within the intervention groups before and after surgery, and the same is summarized in Fig. 3. In all groups there was found to be a statistically significant decrease in FVC in the period between postoperative day 1 and postoperative day 7, when compared to the preoperative period. The FVC values for volume incentive spirometry group almost reached the baseline on postoperative day 7 (p = 1.00) (Table 2).

**Table 2:** Mean difference and percentage change of pulmonary function test values between pre-operative day and post-operative day 1–7 among the three groups.

	Pre-op vs. POD 1	Pre-op vs. POD 2	Pre-op vs. POD 3	Pre-op vs. POD 4	Pre-op vs. POD 5	Pre-op vs. POD 6	Pre-op vs. POD 7
**Forced vital capacity (FVC)** **Mean difference between preoperative day and postoperative days 1–7**							
Flow-incentive spirometry	1.08	0.98	0.91	0.82	0.82	0.67	0.56
% Change	65.06	59.03	54.81	49.39	48.79	40.36	33.73
p value	< 0.001	< 0.001	< 0.001	< 0.001	< 0.001	< 0.001	< 0.001
Diaphragmatic breathing exercise	1.05	0.94	0.90	0.84	0.84	0.74	0.65
% Change	68.38	61.29	58.70	54.83	52.90	47.74	41.93
p value	< 0.001	< 0.001	< 0.001	< 0.001	< 0.001	< 0.001	< 0.001
Volume-incentive spirometry	0.76	0.65	0.55	0.45	0.45	0.26	0.09
% Change	47.53	40.74	34.56	28.39	20.98	16.66	5.55
p value	< 0.001	< 0.001	< 0.001	< 0.001	< 0.001	< 0.001	< 0.001
**Forced expiratory volume in 1 s (FEV1)** **Mean difference between preoperative day and postoperative days 1–7**							
Flow-incentive spirometry	0.83	0.80	0.73	0.65	0.65	0.53	0.44
% Change	59.28	57.14	52.14	46.42	45	37.85	31.42
p value	< 0.001	< 0.001	< 0.001	< 0.001	< 0.001	< 0.001	< 0.001
Diaphragmatic breathing exercise	0.81	0.76	0.70	0.63	0.63	0.53	0.47
% Change	61.65	57.89	53.38	48.12	45.11	40.60	35.33
p value	< 0.001	< 0.001	< 0.001	< 0.001	< 0.001	< 0.001	< 0.001
Volume-incentive spirometry	0.73	0.65	0.57	0.5	0.5	0.22	0.07
% Change	54.47	48.50	42.53	36.56	27.61	16.41	5.97
p value	< 0.001	< 0.001	< 0.001	< 0.001	< 0.001	< 0.001	0.69
**Peak expiratory flow rate (PEFR)** **Mean difference between preoperative day and postoperative days 1–7**							
Flow-incentive spirometry	1.50	1.49	1.32	1.11	1.11	1.00	0.94
% Change	63.02	63.02	55.46	46.63	44.11	42.01	39.49
p value	< 0.001	< 0.001	< 0.001	< 0.001	< 0.001	< 0.001	< 0.001
Diaphragmatic breathing exercise	1.58	1.54	1.42	1.30	1.30	1.21	1.17
% Change	70.08	69.19	63.39	58.03	56.25	54.01	51.78
p value	< 0.001	< 0.001	< 0.001	< 0.001	< 0.001	< 0.001	< 0.001
Volume-incentive spirometry	1.17	1.07	0.94	0.7	0.7	0.39	0.22
% Change	47.95	43.85	38.52	28.68	20.49	15.57	9.01
p value	< 0.001	< 0.001	< 0.001	< 0.001	< 0.005	0.60	0.81

p < 0.001 highly significant, *POD* postoperative day.

**Figure 4:**
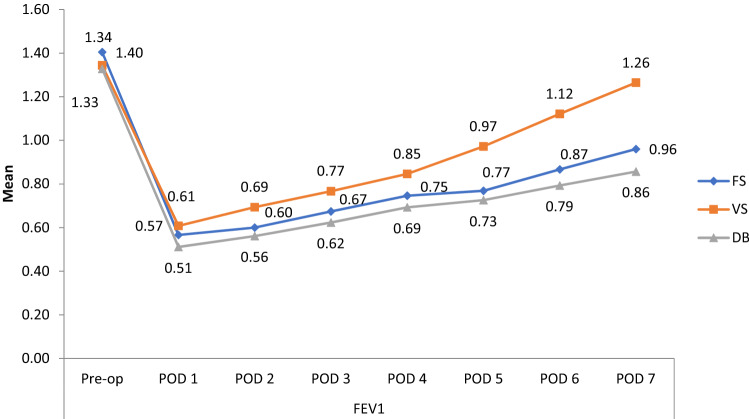
Comparison of forced expiratory volume in 1 s (FEV1) before and after coronary artery bypass graft surgery between three groups.

Forced Expiratory Volume in one second (FEV1) was compared within the intervention groups before and after the surgery and is summarized in Fig. 4. In all groups there was a statistically significant decrease in forced expiratory volume in one second (FEV1) in the period between post-operative day 1 and postoperative day 7, when compared with the preoperative period. The forced expiratory volume in one second values for the volume-incentive spirometry group almost reached the baseline on postoperative day 7 (p = 0.69) (Table 2).

**Figure 5:**
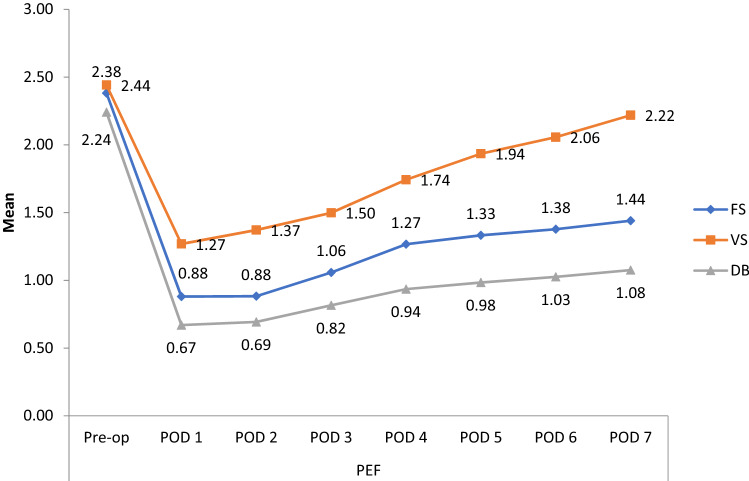
Comparison of peak expiratory flow rate (PEFR) before and after coronary artery bypass graft surgery between three groups.

Peak expiratory flow rates (PEFR) were compared with the intervention groups before and after surgery and are summarized in Fig. 5. In all groups there was a statistically significant decrease in peak expiratory flow rates (PEFR) in the period between post-operative day 1 and postoperative day 7, when compared with the preoperative period. The peak expiratory flow rates values for volume-incentive spirometry group almost reached the baseline on postoperative day 7 (p = 0.81) (Table 2).

**Figure 6:**
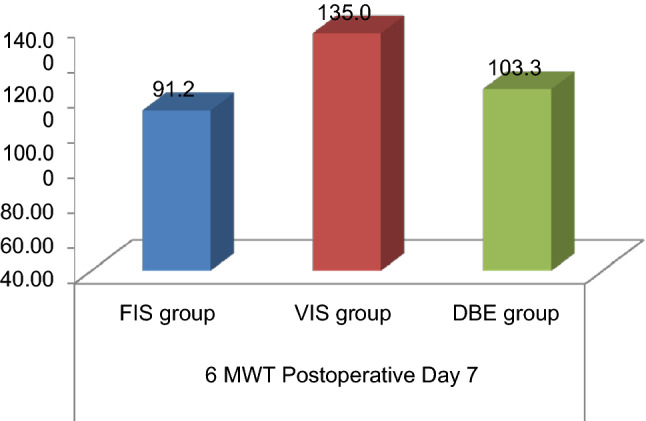
Comparison of 6-Minute Walk Test (6MWT), on post-operative day 7 in the three intervention groups.

The functional capacity i.e. 6-Minute Walk Test (6 MWT) was compared between the intervention groups on postoperative day 7 and is summarized in Table 3. As observed on postoperative day 7, there was a statistically significant increase in the distance walked in the volume-incentive spirometry group as compared to the diaphragmatic breathing exercise group and the flow-incentive spirometry group (p = 0.01) (Fig. 6).

**Table 3:** Comparison of 6-Minute Walk Test, on post-operative day 7, between three intervention groups.

6-Minute Walk Test (6 MWT) (m)	Flow-incentive spirometry group	Diaphragmatic breathing exercise group	Volume-incentive spirometry group
Post-operative day 7 (mean ± SD)	91.25 ± 56.98	103.33 ± 53.30	135.00 ± 51.33

		p value	
**Mean differences compared on postoperative day 7 in between the three groups**			
Flow-incentive spirometry versus diaphragmatic breathing exercise group	12.083	1.000	
Flow-incentive spirometry group versus volume-incentive spirometry group	43.750	0.019	
Volume-incentive spirometry group versus diaphragmatic breathing exercise group	31.667	0.137	

p < 0.001 highly significant.

**Figure 7:**
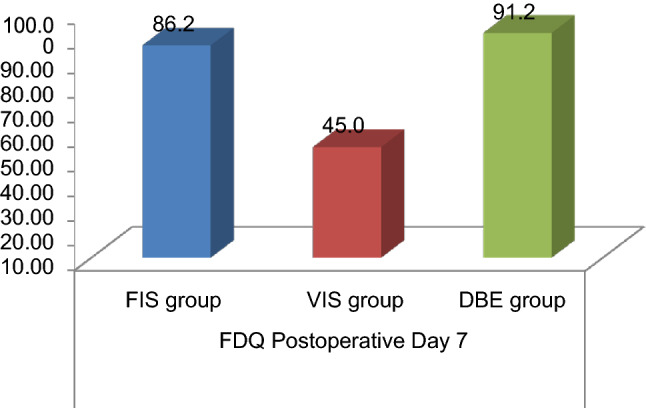
Comparison of Functional Disability Questionnaire post cardiac surgery (FDQ), on the post-operative day 7 in the three intervention groups.

On the postoperative day 7 a comparative evaluation of the functional difficulty questionnaire (FDQ) was made among the three interventional groups. This is summarized in Table 4. Upon comparison among the three intervention groups, a significant improvement was observed on postoperative day 7 in the volume-incentive spirometry group as compared to the diaphragmatic breathing exercise group and the flow-incentive spirometry group (p < 0.001) (Fig. 7).

**Table 4:** Comparison of functional disability questionnaire post cardiac surgery (FDQ), on the post-operative day 7 between three intervention groups.

Functional Disability Questionnaire post (FDQ)	Flow incentive spirometry group	Diaphragmatic breathing exercise group	Volume incentive spirometry group
Post-operative day 7 (mean ± SD)	86.25 ± 21.43	91.25 ± 14.84	45.00 ± 25.88

		p value	
**Mean differences compared on postoperative day 7 in between the three groups**			
Flow incentive spirometry versus diaphragmatic breathing exercise group	5.000	1.000	
Flow incentive spirometry group versus volume incentive spirometry group	41.250	< 0.001	
Volume incentive spirometry group versus diaphragmatic breathing exercise group	46.250	< 0.001	

p < 0.001 highly significant.

In the period between the preoperative day and postoperative days 5 and 7 forced vital capacity (FVC), forced expiratory volume in one second (FEV1) and peak expiratory flow rate (PEFR) were compared among the three intervention groups. The findings are summarized in Tables 5 and 6. There was statistically significant difference among the three intervention groups in terms of forced vital capacity (FVC), the said variables being significantly lower in the flow-incentive spirometry group than in the volume-incentive spirometry group and the diaphragmatic breathing exercise group (Tables 5 and 6).

**Table 5:** Shows the difference between preoperative and postoperative day 5 between the three intervention groups of forced expiratory volume in 1 s (FEV1), forced vital capacity (FVC) and peak expiratory flow rate (PEFR).

Pre-operative day minus postoperative day 5 (mean difference)	Forced vital capacity (FVC) (L)	Forced expiratory volume in 1 s (FEV1) (L)	Peak expiratory flow rate (PEFR) (L/s)
Flow spirometry group versus diaphragmatic breathing exercise group	0.00	0.00	0.2
p value	0.73	0.9	0.49
Flow spirometry group versus volume spirometry group	0.5	0.3	0.5
p value	< 0.001	0.5	0.02
Volume spirometry group versus diaphragmatic breathing exercise group	0.5	0.3	0.7
p value	< 0.001	0.12	0.15

p < 0.001 highly significant.

**Table 6:** Show the difference among the three intervention groups in forced expiratory volume in 1 s (FEV1), forced vital capacity (FVC) and peak expiratory flow rate (PEFR) between preoperative day and postoperative day 7.

Pre-operative day minus postoperative day 7 (mean difference)	Forced vital capacity (FVC) (L)	Forced expiratory volume in 1 s (FEV1) (L)	Peak expiratory flow rate (PEFR) (L/s)
Flow spirometry group versus diaphragmatic breathing exercise group	0.1	0.00	0.2
p value	0.65	0.86	0.49
Flow spirometry group versus volume spirometry group	0.3	0.3	0.7
p value	< 0.001	0.001	< 0.001
Volume spirometry group versus diaphragmatic breathing exercise group	0.2	0.3	0.9
p value	< 0.001	0.001	< 0.001

p < 0.001 highly significant.

## Discussion

In our study we found that Volume-incentive Spirometry improved pulmonary function, functional capacity and functional difficulty in patients who underwent coronary artery bypass graft surgery as compared to flow-incentive spirometry and diaphragmatic breathing exercises.

In our study pulmonary function (FVC, FEV1 and PEFR) showed a decrease on postoperative day 1 on comparison with preoperative values. The pulmonary function values almost returned to baseline on postoperative day 7 as compared to the preoperative values in all the three groups.

Our results are in conjunction with Renault et al., who concluded that there was a mean significant decrease in pulmonary function (FVC, FEV1) after CABG on postoperative day 7 in the flow-incentive spirometry group^3^. Dias et al., found that in the flow-incentive spirometry group there was reduction in pulmonary function (FVC) after CABG surgery on postoperative day 1 which partially normalized by postoperative day 5^18^. Oikkonen et al., found that in the volume-incentive spirometry group after coronary artery bypass graft surgery there was reduction in pulmonary function (FVC, PEF) on postoperative day 1 which recovered (FVC, PEF) on postoperative day 7^19^.

In patients undergoing coronary artery bypass graft surgery the possible reasons for a decrease in pulmonary function (FVC, FEV1, PEFR) during the post-operative period are pain, the effect of anaesthesia, analgesia, insertion of intercostal drains. During the postoperative period, ventilation to the dependent region of the lungs is decreased since the patient performs shallow breathing without intermittent sighs due to the pain associated with median sternotomy and insertion of intercostal drains^3,18,20^.

General anaesthesia may have an effect on the ventilation and lung mechanics, which might lead to reduced ventilation–perfusion, increase in dead space and hypoxemia. Analgesics and other drugs affect the central regulation of breathing and alter the neural drive of the upper airway and muscles of the chest wall resulting in reduced ventilation, decreased sensitivity of the respiratory centres to the stimulation of CO_2_, suppression of the cough reflex and improper production of mucous^19–21^.

Diaphragm dysfunction is caused due to injury to the phrenic nerve due to dissection of the internal mammary artery with electrocauterization and the insertion of chest tubes for pleural and mediastinal drainage after an open heart surgery. This leads to reflex inhibition of the phrenic nerve causing functional disruption of the movement of the diaphragm^3,18–20^.

The possible reason for improved pulmonary function in the volume-incentive spirometry group might be that it is a mechanical device which opens the alveoli and maintains its patency upon the patient’s taking long deep breaths which, again, encourages the patient to breathe until total lung capacity has been achieved. Thereby lung inflation is maintained^5^. Volume-incentive spirometry thus has definite advantages over flow-incentive spirometry since it improves the activity of the diaphragm and reduces the work of breathing. In volume-incentive spirometry, the training volume is kept fixed by the physiotherapist until the maximal inspiratory capacity is achieved. Diaphragm fatigue is minimized since volume spirometry provides a low level of resistance training^22,23^.

Our study is in accordance with Agostini et al. who stated that ‘volume-incentive’ spirometry improves the lung function and also helps in lung re-expansion following CABG surgery^23^. Yamaguti et al., suggested that volume-incentive spirometry and diaphragmatic breathing exercises improve pulmonary function and promote greater diaphragmatic mobility than flow-incentive spirometry^24^. Carvalho et al., stated that the use of ‘volume-incentive’ spirometry promotes early re-expansion of the lungs and also reduces the chances of postoperative pulmonary complications^25^.

The secondary outcome measures in the present study showed that the volume-oriented incentive spirometry group exhibited improved functional capacity demonstrated by the fact that members of the group covered a greater distance in the 6-Minute Walk Test than members of the diaphragmatic breathing exercise group and the flow-incentive spirometry group (mean difference of 35 m). Also, there was an improvement in the scores obtained in the Functional Difficulty Questionnaire (FDQ) by the volume-incentive spirometry group (mean difference = 45 points).

The Six-Minute Walk Test (6MWT) has been validated for use in preoperative and postoperative periods as a measure of recovery and functional capacity in flow- and volume-incentive spirometry groups, members of which have undergone abdominal and thoracic surgeries. Mobilization of postoperative patients with low intensity exercise aims at eliciting cardiopulmonary responses which enhances oxygen transport, assists in reduction of postoperative pulmonary complications and improves the Quality of Life^26–29^. Early mobilization is recommended to patients undergoing coronary artery bypass graft surgery. It has proven to enhance lung volumes and gas exchange^28^. Our results are similar to the findings of Stein t al., who concluded that a 6-day rehabilitation program attenuated the postoperative reduction in respiratory muscle strength and also improved the recovery of functional capacity in the Inspiratory muscle training group after CABG^28^.

Hirschhorn et al., concluded that incorporation of moderate intensity inpatient cardiac rehabilitation improved the distance walked in 6 min by CABG patients on postoperative day 7^29^. Haeffener et al., concluded that flow-incentive spirometry coupled with expiratory positive airway pressure brought about an improvement in the distance walked by patients one week and one month post-CABG^20^.

## Methodology

The study was conducted at KMC Hospital (Ambedkar Circle), Kasturba Medical College, Mangalore, Karnataka India from January 2017–2018. Patients belonging to both genders, age group of 18–80 years, posted for coronary artery bypass graft surgery and referred for physiotherapy by the cardiac surgeon were included in the study. Patients with unstable hemodynamic parameters (arterial pressure < 100 mmHg systolic and < 60 mmHg for diastolic and mean arterial pressure (MAP) < 80 mmHg) having postoperative complications, undergoing invasive mechanical ventilation (IMV) or noninvasive (NIV) for a period exceeding 24 h after admission to the intensive care unit. Those who were cognitively impaired, uncooperative or unable to understand or use the device properly. Patients having vital capacity < 10 mL/kg. Patients with any known pulmonary conditions, undergone recent major cardiac surgery, history of neurological, musculoskeletal disorder and peripheral vascular diseases were excluded. The sample size was calculated based on the values obtained from pulmonary function test in a pilot study (15 subjects, 5 in each group) done by us. The following formula was used for calculating the same;

$$ {\text{n}} = \frac{{2 \times \, \left[ {{\text{Z}}1 - \alpha /2 \, + {\text{ Z}}1 - \beta } \right]^{2 } \sigma^{2} }}{{{\text{d}}^{2} }} $$

whereas the combined standard deviation (0.67), d is the clinically significant difference (0.7), is 2.39 (for 5% significance level for 3 groups), is 0.84 at 80% power. The sample size was 24 in each group (total 72 subjects).

### Procedure

The study protocol was submitted for approval from the Scientific Committee and the Institutional Ethics Committee Kasturba Medical College, Mangalore (IEC KMC MLR 11-17/237). Upon approval from the Committees, the study was registered for Clinical Trial Registry (CTRI/2018/01/011324) Reference number (REF/2018/01/016739) link (http://ctri.nic.in/Clinicaltrials/rmaindet.php?trialid=22202&EncHid=46114.87480&modid=1&compid=19). Date of first enrollment: 20/01/2018. All methods were performed in accordance with the relevant guidelines and regulations stated by the Institutional Ethics Committee Kasturba Medical College, Mangalore. The subjects were then selected based upon the inclusion and exclusion criteria, the purpose was explained and the subjects consenting to participation were included in the study. A written informed consent was signed by the participants who initiated a voluntary participation. The patients were divided into three groups:

 − Flow-oriented incentive spirometer group
 − Volume-oriented incentive spirometer group
 − Diaphragmatic breathing exercises group

Sequencing was done using a computer generated random numbered tables. Block randomization was done. The participants will be divided into 12 blocks with 6 participants in each block. Allocation was done using sequentially numbered, opaque sealed envelopes. Group information was concealed in a sealed opaque envelope and was revealed to the patients only after they are recruited into the treatment group by an investigator who was a physiotherapist. The inpatient cardiac rehabilitation program was delivered to the patients by another investigator who is also a physiotherapist and was blinded to allocation and also to the assessment of outcome measures. The outcome measures were assessed by the primary investigator.

Following the allocation to groups, the patients in all the three treatment groups were visited one day prior to surgery. Pre-operative information was offered and based on his/her group; flow-oriented incentive spirometer, volume-oriented incentive spirometer or diaphragmatic breathing exercises were taught to each patient. Use of ventilatory devices (i.e. either flow-oriented incentive spirometer/volume-oriented incentive spirometer/diaphragmatic breathing exercise-as per the group allotted), airway clearance techniques (huffing, coughing, mucous clearance devices), circulation (ankle toe movements, upper limb and lower limb range of motion exercises), thoracic expansion exercises, mobilization (edge of bed sitting, walking, stair climbing) were provided.

**Figure 8:**
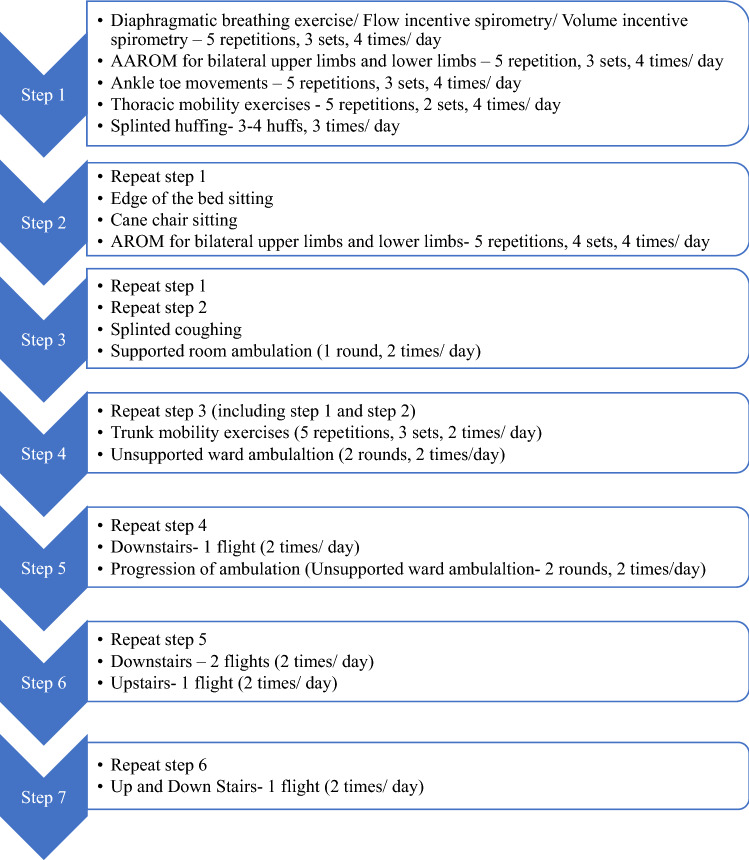
Phase 1 cardiac rehabilitation program (stepwise protocol for 7 days until discharge).

A standard 7-day Inpatient Cardiac Rehabilitation protocol was taught to the patient^15,16^ (Fig. 8).

Pulmonary function tests (PFT) measured the following variables: forced vital capacity (FVC), forced expiratory volume in the first second (FEV1), peak expiratory flow rate (PEFR).These were taken on the preoperative day and on the 1st, 2nd, 3rd, 4th, 5th, 6th and 7th postoperative days, for all the groups. These measurements were taken by the primary investigator.

The 6-Minute Walk Test (6MWT) was performed according to the American Thoracic Society’s guidelines^17^. The 6-Minute Walk Test was taken on the day of discharge, for all the groups. These investigations were taken by the primary investigator.

#### Pulmonary Function Test

Pulmonary Function Test procedures were carried out according to the American Thoracic Society/European Respiratory Society guidelines. The following variables were recorded: forced vital capacity (FVC), forced expiratory volume in the first second (FEV1) and peak expiratory flow rate (PEFR). The best value of the three tests was taken^5^.

#### 6-Minute Walk Test

The 6MWT was performed according to the American Thoracic Society’s guidelines. Participants were instructed to walk as far as they could along a 30-m corridor for six minutes with the aim of achieving their maximum possible walking distance in 6 min time. Standardized instructions were provided each minute. Subjects were permitted to stop and rest if required but were encouraged to continue walking as soon as they were able to do so^17^.

Heart rate (HR), respiratory rate (RR) and oxygen saturation (SPO_2_) were measured using pulse oxymeter; dyspnea (modified Borg scale) and fatigue were measured at the beginning and immediately after the test. The outcome of the test was the covered distance: the longer the distance covered by the patient, the better their functional performance^17^.

The Functional Difficulties Questionnaire (FDQ) is a paper-based questionnaire that contains generic instructions for completion. The Functional Difficulties Questionnaire (FDQ) comprised 13 separate functional tasks which are necessary in everyday life and involve movements associated with the thoracic region. These tasks included upright sitting, walking with arms swinging freely, coughing/sneezing, rolling over in bed, getting out of bed, washing hair, scratching the back, picking up an object off the ground, turning to reach backward, doing the clasp of a bra or tucking in a shirt at the back of pants, putting on a dressing gown/cardigan/jacket, drying the back with a towel, and pushing a set of drawers shut^9^.

**Figure 9:**
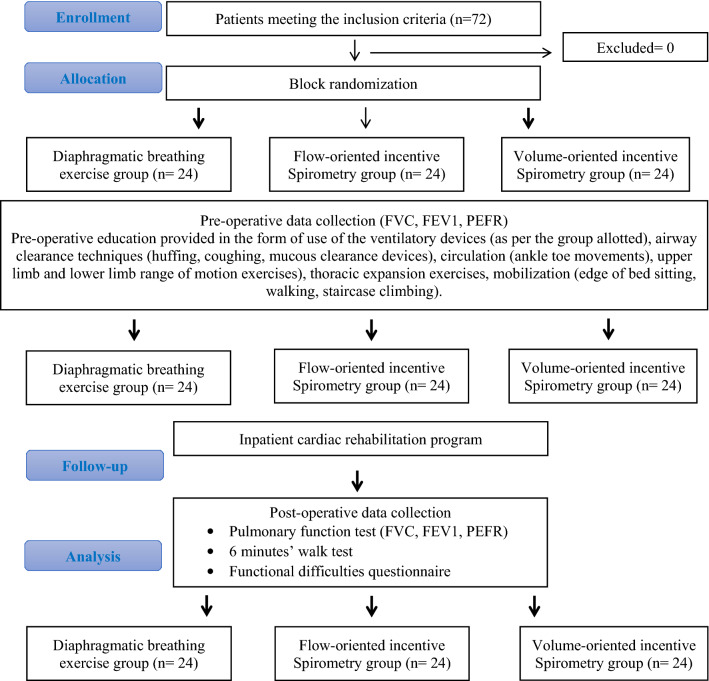
CONSORT flow diagram.

The FDQ was administered to patients on the day of discharge. The FDQ is a paper-based questionnaire that includes generic instructions for completion. After reading these, on unmarked 10-cm visual analogue scales (VAS), participants can grade 13 different tasks according to the level of difficulty experienced when completing each task. The VAS end points were "No difficulty" and "maximum difficulty". In accordance with a standardized administration procedure, the therapist was present to aid with the execution of the questionnaire. Individual VAS scores, measured to the nearest millimeter, were then aggregated to form a potential number of 130, with higher scores reflecting greater functional difficulty encountered. If a given task could not be accomplished by the participant, it was automatically graded as a 10 to reflect maximum difficulty. These investigations were supervised by the primary investigator. The CONSORT flow diagram is mentioned in Fig. 9.

### Methods to Perform Flow-Oriented Incentive Spirometry (FIS) and Volume-Oriented Incentive Spirometry (VIS):

The patient was placed in the half lying position (45°), with a pillow under the knees. The patient was instructed to inhale with a slow and deep sustained breath, holding it for a minimum of 5 s and then was asked to exhale passively in order to avoid any forceful expiration. Initially, the patient was given a demonstration and then was asked to perform the procedure. The patient was instructed to hold the spirometer upright and to perform flow-oriented incentive spirometry by inhaling slowly which would lead to raising the ball in the spirometer. The same procedure was followed for the volume-oriented incentive spirometry, but the patient was asked to inhale in order to raise the piston or plate in the chamber to the set target^5^.

The patient was instructed to perform 3 sets of 5 repeated deep breaths. This was performed by the patient every waking hour. The therapist administered the exercise four times a day and the patient was instructed to perform the same for the rest of the day. The patient was asked to keep a record of the exercises performed by him/her by entering these in a log book which was provided beforehand^5^.

### Method to perform diaphragmatic breathing exercise (DBE)

The patient was asked to sit in a half lying position (back and head were fully supported and abdominal wall was relaxed) and diaphragmatic breathing was performed. The therapist placed her hands just below the anterior costal margin, on the rectus abdominus muscle, while the patient was be asked to inhale slowly and deeply through the nose, with a three second inspiratory hold from the functional residual capacity to total lung capacity. The patient was then instructed to relax the shoulders and keep the upper chest quiet so that the abdomen may be a little raised. The patient was then asked to exhale slowly though the mouth^5^.

The patient was asked to experience a slight raise and subsequent fall of the abdomen by placing his/her hand below the anterior coastal margin, during inspiration and expiration. The patient was asked to perform 3 sets of 5 deep breaths, with the therapist administering them four times in a day, and the patient was instructed to perform the same every waking hour for the rest of the day. The patient was asked to breathe normally, in between the repetitions of the diaphragmatic breathing exercise. The patient was asked to keep a record of the exercise performed by entering it in the log book provided beforehand^5^.

### Statistical analysis

Data was analyzed using IBM SPSS package version 25.0. Armonk, NY: IBM corp. ANOVA and post hoc analysis (Bonferroni’s *t*-test) were carried out to verify the within-groups differences. Between groups differences were compared using two-factor ANOVA.

### Limitations

The duration and type of anaesthesia and analgesia were not recorded and this could probably affect the outcomes. The patients were provided with log books in which to make an entry every time they performed the technique. This was meant to enable the researcher check their adherence to the prescribed regimen. But there was no way to verify the authenticity of these entries.

### Future research

Future studies can be carried out in a similar way with a large sample size, on patients who have undergone valve replacement surgeries. Future research on the use of alternative techniques can be carried out by assessing respiratory muscle strength and the comfort of patients who have undergone cardiac surgery. The effect generated by a combination of diaphragmatic breathing exercise and incentive spirometry can be studied in cases of thoracic and abdominal surgeries.

### Clinical implication

Volume-oriented incentive spirometry can be recommended for all the patients postoperatively and preoperatively over flow-oriented incentive spirometry and diaphragmatic breathing exercise as an intervention for the generation and improvement of pulmonary function, functional capacity and functional difficulty questionnaire (FDQ) in the management of coronary artery bypass graft surgery. For general CABG population, family members need to assist the patient in this regimen of inpatient cardiac rehabilitation for better recovery.

## Conclusion

- The conclusion obtained from our study is that in patients undergoing coronary artery bypass graft surgery there is decrease in the pulmonary function (FVC, FEV1, PEFR) in all the three intervention groups from postoperative day 1 to postoperative day 7, when compared with preoperative values.
- Pulmonary function values (FVC, FEV1, PEFR) reached baseline on postoperative day 7, and functional capacity and functional difficulty questionnaire (FDQ) showed an improvement in the volume incentive spirometry group.
- From the present study we concluded that Volume-oriented Incentive Spirometry proved to be beneficial in improving pulmonary function (forced vital capacity—FVC), functional capacity and functional difficulty questionnaire (FDQ) when compared to flow-oriented incentive spirometry and diaphragmatic breathing exercises.

## Data Availability

The data used to support the findings of this study are available from the corresponding author upon request.

## Abbreviations

VIS: Volume-oriented incentive spirometry
FIS: Flow-oriented incentive spirometry
DBE: Deep breathing exercise
PFT: Pulmonary function test
FVC: Forced vital capacity
FEV1: Forced expiratory volume in 1 s
PEFR: Peak expiratory flow rate
6MWT: 6 Minute Walk Test
FDQ: Functional Difficulties Questionnaire
